# Mental Health and Wellbeing in Cohousing Communities: An Ethnographically Informed Approach

**DOI:** 10.1002/jcop.70100

**Published:** 2026-04-09

**Authors:** Jake Maxwell Watts, Terry Hanley, Erica Burman

**Affiliations:** ^1^ University of Manchester Manchester UK

**Keywords:** cohousing, community, ethnography, housing, intentional communities, mental health, wellbeing

## Abstract

Loneliness and social isolation are growing public health concerns. Conversely, community membership is associated with reduced mortality risk and ameliorates mental distress. This paper explores how residents of a self‐governing cohousing community, comprised of private dwellings and common spaces, understand the impact of community living on their mental health and well‐being. An ethnographically informed method is used. Data is analysed using reflexive thematic analysis and comprises fieldnote observations of a community, semi‐structured interviews, and relevant community documentation. Four themes were generated: (1) Social Pollination: How a Community Protects, (2) Finding Your Tribe: Meaning and Purpose in Cohousing, (3) Deconstructing Defensible Spaces, (4) Conflict and Tension in a Social World. Residents benefitted from a strong sense of meaning and identity, support when experiencing distress or raising children, and increased security from a sharing economy. Perceived social burdens, conflict, and distress are experienced and mitigated by resolution processes.

## 1 Introduction

It is difficult to look at the picture of mental health globally and not feel concern. Between 1990 and 2019, the number of disability‐adjusted life‐years (DALYs) lost due to mental disorders increased from 80.8 million to 125.3 million (GBD 2019 Mental Disorders Collaborators 2022), made worse still by the COVID‐19 pandemic (Santomauro et al. 2021). Mental illness is one of the largest causes of disability, costing an estimated 418 million DALYs, or about US$5 trillion annually in 2019 (Arias et al. 2022). Young people are particularly at risk. In the United Kingdom, rates of anxiety, mood, and other disorders increased substantially between 2003 and 2018, and are not explicable solely by common interpretations such as changes to diagnostic criteria, reduced stigma, and increased awareness (Cybulski et al. 2021). This is despite a paradoxical increase in overall provision of care (Skinner et al. 2022). Some argue that global megatrends are to blame, such as rising intergenerational inequality, unregulated social media, wage theft, insecurity of employment, and climate change (McGorry et al. 2024). One key factor that this paper argues warrants further attention is that of community and social connection.

Humans are a profoundly social species. We evolved to depend on each other for resources, protection and reproduction, facilitated by language (Dunbar 1993). Our social interactions determine brain development, and our risk of mental illness is influenced by our position within a social network (Lamblin et al. 2017; Meyer‐Lindenberg and Tost 2012). We are more likely to thrive when we are strongly connected to others. Sadly, the inverse is also true: when socially isolated (cut off from others) or lonely (a subjective deficit in connection), we suffer significant mental and physical consequences. Loneliness can increase the risk of a premature death by 26%, social isolation by 29%, and living alone by 32% (Holt‐Lunstad et al. 2015) – levels comparable with well‐documented clinical risk factors such as smoking (Pantell et al. 2013). Loneliness and social isolation are also associated with overall poorer mental health outcomes. Given the psychological and physical consequences, it is concerning that unprecedented numbers of people are living alone (Surkalim et al. 2022). Most pre‐industrial settlements had fewer than 5% single‐person households; now at least a third live alone across most of Europe, the United States and the United Kingdom (Snell 2017).

This paper explores the experiences of groups of people who have consciously chosen to create neighbourhoods that maximise the opportunities for interaction and connection. These spaces, often comprised of a few dozen people or more, are a type of intentional community known as ‘cohousing’, which originated in Denmark in the 1960s (McCamant and Durrett 1988). While humans have lived together in communities across time and cultures (McNeill 2024), modern cohousing in this tradition is growing in popularity. Most definitions describe self‐governed communities comprised of private dwellings situated around shared spaces such as a multi‐function ‘common house’. In the UK context, the focus of this paper, a local members' organisation counts more than 20 established cohousing communities in the country (UK Cohousing Network 2023) and more than 65 in development. Communities are run collectively, utilising consensus decision‐making, with shared responsibility for maintenance, common spaces, and formalised agreements about values and expectations. Communities aim to maximise opportunities for interaction and self‐select their members (Chiodelli and Baglione 2014). There is significant variation in how such communities are managed financially – in the United Kingdom, most are comprised of owner‐occupier dwellings, and all homeowners also contribute to the site running costs in the form of a service charge. In many cases, communities aim to share facilities, such as a laundry room, toolshed, gardens, workshop and parking/car use, in order to bring down individual living costs through economies of scale. The participant community in this study, Poplar Grove, pioneered a unique financial model that provided additional benefits such as a group mortgage, allowing better rate negotiation and for mortgage repayments for residents earning less (and therefore paying back principals over longer) to be supported by residents paying back their premiums faster, thereby more easily satisfying lender requirements.

While social isolation and loneliness are risk factors for poor health outcomes, receiving social support and maintaining regular interaction with others can be beneficial, reducing all‐cause mortality risk (Becofsky et al. 2015). Symptoms of depression are reduced by social support, while loneliness and small network size are associated with increased depression symptoms and anxiety disorders (Wickramaratne et al. 2022). In times of acute stress, social fragmentation can increase the risk of post‐traumatic stress disorder (Bryant et al. 2017), and individuals with a strong sense of community belonging have increased odds of better mental health (Allen et al. 2021; Palis et al. 2020). Theoretical models posit that benefits are created by promoting (1) health behavioural pathways such as diet and exercise, (2) psychological pathways such as self‐esteem and coping, and (3) physiologic pathways by mitigating the negative effects of chronic stress on immune system and cardiovascular function (Berkman et al. 2000). Group membership is seen as a way of meeting needs, allowing people to co‐regulate, providing opportunities, and creating a secure base. However, not all interaction is seen as positive for mental health, especially in the presence of conflict, the perception of threat or rejection, or where there are unsupportive connections (Andersen et al. 2021).

Existing research on the psychological effects of living in a cohousing community is limited and focused primarily on psychosocial factors. Cohousing is associated with reduced loneliness, higher trust in neighbours, and a strong sense of belonging (Scanlon et al. 2021). Residents surveyed during the COVID‐19 pandemic in Germany reported lower levels of depressive, anxious, and compulsive or eating‐disordered behaviours than a control group and used fewer unhelpful coping strategies such as avoidance (Schetsche et al. 2020). A recent systematic review found broadly positive associations between cohousing residency and good physical, mental health, quality of life, and wellbeing, although the authors warned that extreme caution should be exercised in drawing conclusions due a dearth of quality data and methodological limitations (Carrere et al. 2020). A separate scoping review found indications that cohousing residency is associated with higher social capital (Warner et al. 2024).

To summarise, existing mental health interventions have struggled to address rising rates of loneliness, social isolation, and psychological distress in high‐income countries such as the United Kingdom, even as mental health services expand. This paper explores whether cohousing could offer a promising non‐clinical and holistic method of addressing the problem that complements existing services. Research to date implies a positive impact, but it is rarely from the UK context, is of limited quality and scope, and generally does not explore any negative impacts on mental health and wellbeing.

Given the breadth of psychological and psychosocial factors explored in previous cohousing research, this research employs an expansive definition of mental health beyond diagnostic criteria (Galderisi et al. 2015). Common definitions of wellbeing tend to be broad (Levy 2014), encompassing factors such as quality of life, meaning, and purpose, and are determined by social, economic and environmental conditions (World Health Organization 2025). Most applicable to this research is the definition of wellbeing by Atkinson et al. (2020), which distinguishes three kinds: (1) variables affecting individual lives, such as how a person feels about their house or job, (2) variables affecting collective living, such as how people feel about local transport, activities, or social factors like trust, and (3) variables capturing community wellbeing as more than the sum of its parts – that is, being well together, through assessments of factors such as community cohesion, shared values, belonging and collective mood.

Using those definitions of the variables at hand, this research is guided by the following question: *How do residents of one cohousing community in the UK understand and experience the impact of community living on their mental health and wellbeing?*

## 2 Materials and Methods

### 2.1 Selection of Methods and Study Design

A qualitative design was chosen based on ethnographic methods. Ethnography is experiencing a resurgent interest for its potential to capture culture, context, process, and provide contextual understanding in complex communities (Case et al. 2014), including in the field of psychology (Simanjuntak et al. 2022; Suzuki et al. 2005). Consistent with modern adaptations, this study compresses fieldwork to a period of 2‐weeks' intensive immersion in a single community.

### 2.2 Ontology and Epistemology

As this study sought to understand the nature of both individual and collective experiences in community life, it was appropriate to take a relativist ontological stance, which holds that reality is both constructed by individual perception *and* socially constructed through the interaction of people within cultural contexts. This stance is particularly relevant to understanding communities, because it implies that concepts such as mental health and community are socially constructed phenomena that mean different things to different people (Mertens 2020). A social‐constructionist epistemological stance was therefore suitable, which assumes that knowledge is built from the bottom up through observations of the world and interactions between researcher and participant, as opposed to a deductive approach that seeks to test a hypothesis aligned with a pre‐conceived theory (Ponterotto 2005).

### 2.3 Sample and Recruitment

Purposive sampling was used to identify one community, referred to by the pseudonym ‘Poplar Grove’. Access negotiations were completed with community gatekeepers. Poplar Grove consists of approximately 20 dwellings plus a common house, located within a UK urban environment. Private homes are distributed around a pedestrianised communal outdoor space with seating, gardens, allotments and a children's play area, and outbuildings provide shared facilities such as bike sheds and a workshop. The common house includes a large kitchen and dining area for communal meals, a multi‐function living space, shared offices/guest bedrooms, and a post room.

Nine interviewees were recruited during fieldwork using purposive sampling. A cross‐section of the community was sought to include a diverse range of perspectives and demographics. Five interviewees were male and four female, with an average age of 48.2 years (range: 18–72 years). Four were living in family houses with children, four alone, and one with a partner. Seven were residents at the community since its inception 11 years prior, one for 10 years, and one for less than a year. Eight were educated to degree level or above. Six were identified as middle‐class, while three were identified as working or lower‐middle class. Informed consent was gained from the community as a whole and from individual interviewees.

### 2.4 Data Collection

A 2‐week residential period of fieldwork was agreed. Data collection, between April and June 2024, consisted of daily fieldnotes recording observations and events experienced by the on‐site researcher, and nine in‐person interviews with residents. Fieldnotes ranged from approximately 300–1000 words per day and kept an account of events, persons and places experienced by the researcher. Ethnographic note‐taking is a subjective process – while fieldnotes are faithful representations of real events, they inevitably involve choices (such as whether to record something or not) that may not always be obvious (Madden 2017), which is why a reflexive diary was kept by the researcher (see Section 2.6 below).

Interviews were semi‐structured, with key topics informed by a review of the literature to facilitate the pursuit of themes of interest while also allowing interviewees some freedom to contribute insights not previously considered. The interview schedule was designed to balance broad ‘opening’ questions with topic‐specific items, and was iterative – later interviews included questions informed by earlier interviews.

Documentation pertaining to community vision, values, and processes/agreements was obtained. The researcher kept a daily journal to aid in reflexivity that was not included in the subsequent analysis. Interviews were semi‐structured, lasting approximately 60 min each. The interview schedule was informed by prior research on the topic and consisted of approximately 10 questions focused on subjective experiences of mental health and wellbeing in cohousing (e.g. ‘*Overall, how would you describe the impact that community living has had on your mental health and wellbeing*’ and ‘*To what extent have you felt lonely and/or socially isolated in your life and how has community living affected that?*’).

### 2.5 Qualitative Analysis

Data analysis was conducted using reflexive thematic analysis (RTA) according to Braun et al. (2022). Coding analysis was completed on interview and fieldnote data using NVivo 14. Documentary data and reflexive notes were used for cross‐referencing and facilitating understanding of community processes, but were not coded directly for analysis. Interview data were transcribed manually using an orthographic transcription style (Braun and Clarke 2013). RTA sees the researcher as a storyteller – the stories told about the data are inevitably shaped by the researcher's personal positioning, lived experience, assumptions and expectations about a topic (Braun et al. 2022). It follows a six‐step iterative process outlined by Braun et al. (2022) involving (i) familiarisation with the data, (ii) coding, (iii) initial theme generation by clustering similar codes to explore patterns, (iv) reviewing and developing themes to capture meaningful patterns, (v) refining, defining and naming themes that each tell an interpretative story about the data, and (vi) writing the report.

### 2.6 Quality Criteria and Reflexivity

Researcher transparency and reflexivity are essential components of quality and trustworthiness in qualitative research (Nowell et al. 2017), particularly where the researcher is also a participant in the generation of knowledge (Cardano 2014). The lead researcher and sole fieldworker is a white, male, millennial British student, conducting this research as part of a Counselling Psychology professional doctorate. He had some pre‐existing personal experience of intentional communities, having lived in one for 4 years as a young child, and retains fond memories but no adult experience of communal living and no association with the participant community.

To ensure credibility and trustworthiness, a 15‐point quality checklist for RTA was applied (Braun and Clarke 2006), and where possible, in theme generation, multiple sources of data (observation, interviews, documents) and cross‐checking ideas between interviewees were used. Detailed reflexive notes were kept throughout fieldwork and analysis to assist in identifying biases and assumptions. An ethnographer may encounter choices and dilemmas at all stages of the research, and therefore reflexivity is required at each point along the way (Roberts and Sanders 2005). Reflexive notes included reflections on the emotional and cognitive experience of conducting participatory research, and were discussed at length with research supervisors to bring to awareness any issues regarding bias or positioning.

## 3 Results

**Figure 1:**
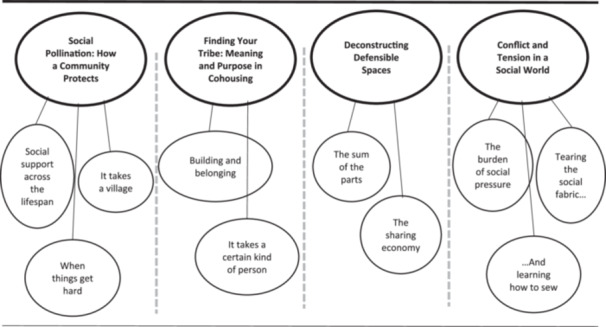
Themes and sub‐themes.

Four themes were generated, comprised of 10 sub‐themes illustrated in Figure 1, generated from 800 unique codes.

### 3.1 Theme 1 – Social Pollination: How a Community Protects

Residents said that community social interactions facilitated by cohousing appear to be a protective factor for mental health and well‐being by providing both emotional and practical support during life events and across the lifespan. One participant coined the term ‘social pollination’ to compare the small daily encounters between residents to pollinating a garden.

#### 3.1.1 Social Support Across the Lifespan

The frequency and quality of social interaction were described as overwhelmingly positive, fending off loneliness, and creating a sense of identity and belonging. Poplar Grove culture was described as a ‘rolling economy of favours and support’ (Interviewee 7), and social interaction was both planned, such as communal meals, and spontaneous, such as encounters in shared spaces. Several interviewees stated they felt supported by having regular social interaction with like‐minded people who could take part in their lives, understand them, and meet their needs, even when they do not know they need them. Other members said they had only realised how previously socially isolated or lonely they were after moving:

‘You don't always recognise that you are lonely … you may fill your evening with watching TV or drinking a bottle of wine or doing this that and the other and and you think everything's okay … it's only when you are in a different – break out of that situation and spend time with people that you that I can look back and say I think I was definitely lonelier in [my] previous [life] than I am living here’.

(Interviewee 3)

#### 3.1.2 It Takes a Village

Poplar Grove is home to several children (age range: 0–18) who are given free rein in the physical environment, including outdoor spaces and the common house. Raising children was generally experienced by members as a collective endeavour involving information‐ and resource‐sharing. All parent members of the community saw cohousing as broadly beneficial for raising children, particularly due to the support available when a child is born:

‘My second child, his initial childcare was all done here. We shared a nanny with two other families. And it was just a fantastic environment to, yeah, it's really easy … my experience with maternity leave was really different. And just being able to walk out my door and see another adult, sometimes not necessarily even knowing that I needed that, was a really different experience’.

(Interviewee 6)

In one case, where the parents of two children had split up, the two adults moved into separate apartments within the community, allowing the children to retain continuity in their environment and friends. Other parents said they most valued logistical support from community members with tasks like school pick‐up and meal preparation. An older resident said her own child had moved abroad, and she felt being around other people's children had been supportive of her mental health.

#### 3.1.3 When Things Get Hard

Community members were not immune to mental health difficulties. Several residents disclosed struggles, including depression and bereavement. Most described the community as protective. However, it was clear from one case of sexual abuse experienced by a child in the community that a large social environment carries its own risks, and different perspectives on how to manage challenges could cause friction. Some members said they had grappled with the extent to which the community should be responsible for safeguarding and tending to the care and emotional needs of individual members.

Several interviewees spoke of the community as being supportive of their mental health during times of stress, such as bereavement:

‘I think, if I wasn't here … [this] could have been a period in my life where I would have become quite lonely, quite reserved, reflective, withdrawn. Yeah, maybe even kind of depression. That hasn't really happened’.

(Interviewee 5)

Despite interviewees generally seeing the community as a protective influence, some expressed frustration with the complexities of consensus decision‐making and compromise. In the case of the COVID‐19 pandemic, this appeared to cause ruptures and put additional strain on mental health and well‐being:

‘Some people were incredibly anxious about it. And some people weren't. And so agreeing things by [expletive] consensus was hard work and painful and, it meant that too often, it felt too often, finding agreement on the lowest common denominator, which is pretty restrictive. So I think that yeah, that dissolved certainly some of the trust and community glue’.

(Interviewee 9)

### 3.2 Theme 2 – Finding Your Tribe: Meaning and Purpose in Cohousing

The data suggests that the act of building and maintaining a cohousing community, through all the challenges that it presents, serves to bring people together with a sense of meaning and common purpose, thus underpinning wellbeing and mental health.

#### 3.2.1 Building and Belonging

The struggle of starting a community – and navigating the financial hurdles of a housing market engineered for private home ownership – appeared to have created a sense of shared adversity that had brought members together. Celebration of this achievement was recorded in fieldnotes:

‘I was showed a photo album of the construction process – it depicts residents sharing their ideas for the community on Post‐it notes, including intentions and vision for priority themes, such as connection with neighbours … children are involved, helping make the walls and babies are asleep amongst all the paperwork’.

The newest adult member said that he was attracted to the community because of progressive ideas about home ownership, environmental sustainability, and affordability:

‘People here are exploring quite interesting ways of living that are like very progressive … I guess it's a kind of antithesis to the kind of private housing market. How do you create settlements that aren't just based on speculation or kind of, I guess, asset wealth accumulation? Then how do you answer the question of kind of some form of resistance to climate change, or indeed, like reducing your impact on the planet? So there's a really kind of interesting kind of integration of those things’.

(Interviewee 5)

#### 3.2.2 It Takes a Certain Kind of Person

While many community members said they valued diversity and plurality, Poplar Grove is fairly homogenous in character. Some said this due to a selective process for new members, but also a requirement that members sign up to the collective vision and values and commit to a communal way of life that requires certain social skills and beliefs that may not suit everyone:

‘I think you need to be someone who is fairly sociable, and who can manage to navigate quite a large number of relationships with people. I think you need to be flexible’.

(Interviewee 6)

In one case, an interviewee said he felt culturally different from others and that his opinions on politics and the environment felt unwelcome. Some said they felt pressure to conform to a community culture. Interviewee 5, for instance, worried he was ‘not eco‐enough’, while Interviewee 1 said he sometimes felt guilty about using shared cars.

### 3.3 Theme 3 – Deconstructing Defensible Spaces

Poplar Grove, in deconstructing the traditionally ‘defensible’ private spaces of the outside world that exist behind locked doors and fenced gardens, has sought to create a quality living space with facilities that would not otherwise be available. Residents said this creates a sense of security and economic opportunity that is beneficial for mental health and well‐being.

#### 3.3.1 The Sum of the Parts

Residents said communal spaces provided opportunities for socialising and activities that would not have been affordable otherwise, like allotments or play areas. The common house, with elevator access, multi‐function space and a communal kitchen, is used for parties and events, and creates a centre of gravity for socialising. Although some residents felt they sometimes experienced social fatigue and valued their own private spaces, many said that the facilities offered by the shared spaces were far more than they could have afforded on their own, including green spaces and pedestrianised spaces:

‘I think the the actual physical design of the complex is, like transformational compared to like, a road running right in front of your front door, very little garden space, nowhere to do any activities nowhere to grow’.

(Interviewee 5)

#### 3.3.2 The Sharing Economy

Residents said a culture of sharing and a conscious decision to decouple from housing prices allowed the community to adopt a financial model that provides access to a quality of life that would not normally be affordable, boosting wellbeing and supporting mental health. Poplar Grove's financial model allows residents to join a collective mortgage scheme that requires individuals to make repayments proportionate to their wages, so those on lower incomes pay off their debt at a slower pace. House prices are pegged to wage indices to remain affordable in perpetuity, but residents do not benefit from appreciation in the commercial market. A range of mortgage repayments remains sustainable provided the community maintains an overall level of income to meet collective minimum repayments demanded by their lender. The substantially lower living costs at Poplar Grove allow many individuals to work part‐time and spend more time with family and in leisure activities:

‘The housing isn't expensive, so I don't have to work full time. And historically, there's been a low proportion of people working full time … I think probably now there's less than less than a handful of people who work full time’.

(Interviewee 9)

### 3.4 Theme 4 – Conflict and Tension in a Social World

Many residents spoke about social conflict and workshare tensions, which some said had caused them to feel stress about their place in the community and affected their mental health and wellbeing.

#### 3.4.1 The Burden of Social Pressure

Several community members said they felt burdened by the amount of work, such as mandatory participation in tasks like maintenance and administration. Interviewees said they put pressure on themselves to contribute more, leading to burnout or resentment:

‘There's a certain amount of anxiety and guilt and overwhelm that comes with being here as well … I can feel quite triggered by just like the pressure to be pulling your weight and be involved and just be like present’.

(Interviewee 7)

Others said they felt judged when not conforming to the majority culture, especially around environmentalism:

‘I'm probably the least sustainable person here, because I'm very much a fashion girl. So I think I get quite judged’.

(Interviewee 8)

A few participants noted that managing the community felt like a form of employment, involving dual relationships with neighbours as both friends and colleagues. They said this could create a mental load and a sense of not being able to escape from the pressure to be productive, and that consensus decision‐making could be burdensome.

#### 3.4.2 Tearing the Social Fabric

Interpersonal conflict was a feature of life at Poplar Grove, and due to the close‐knit nature of cohousing living arrangements, its impact could be felt keenly. Disputes appeared to be managed amicably, although some people spoke of permanent ruptures and an impact on all residents:

‘If there's a real conflict between a couple of people, then that feels more upsetting and dangerous than it does if it was out in the community, because I'm always concerned about the community remaining a happy, healthy place to live. It worries me when there's conflict’.

(Interviewee 2)

Some residents noted (sometimes in a self‐mocking tone as below) that the social consequences of falling out had led them to catastrophise, and spend time worrying about their place in the wider group:

‘I'm going: “this is not working with this person. We're falling out of this issue. Oh, God isn't working. Oh, God, we're going to fall out what if we bump into each other? What if we argue? What then if I have to move out?”’.

(Interviewee 1)

#### 3.4.3 And Learning How to Sew

Rupture repair is designed into the way Poplar Grove operates and acts to mitigate the mental health and well‐being impact of conflict. For example, an agreement outlining workshare expectations from members had been recently implemented. Community members said other processes, such as conflict mediation, were often used and had been broadly effective. Several residents said that they expected occasional ruptures, and that getting good at resolution is more important than conflict‐avoidance:

‘I think the only thing that you can do is build‐in processes to support people through the difficult moments … you know, there will always be clashes between people. And, yeah, how do you deal with that, as the community is probably more important, I think that you can prevent it’.

(Interviewee 6)

## 4 Discussion

### 4.1 Cohousing as Beneficial for Mental Health and Well‐Being

This study aims to understand how people living in cohousing communities understand and experience their mental health and wellbeing. Findings are consistent with previous research indicating that cohousing likely has a positive overall impact (Carrere et al. 2020; Scanlon et al. 2021), acting through predictors of wellbeing such as meaning‐in‐life and social support (Kehl and Then 2013) and by residents providing active, practical and emotional support to each other (Grinde et al. 2018). Findings show how cohousing can play a stress‐buffering role during negative life experiences and mental health challenges, which is consistent with theoretical models of community (Berkman 1995; Cohen and Wills 1985), and residents reported benefits to their mental health and wellbeing through affordability and access to shared spaces.

The results provide an account of the specific ways in which mechanisms such as social support operate at the interpersonal and community levels over time. They suggest that cohousing can play a mediating role in loneliness and as a supportive function across the lifespan, such as for parents and older adults. For example, existing evidence indicates that sharing childcare with non‐parental adults can be beneficial (Kesselring et al. 2016). In the cohousing context, this is facilitated by a built environment that includes spaces for children and by community values around intergenerational interactions. This adds to the current literature, most of which provides only a snapshot measure of wellbeing or mental health based on quantitative rating scales or by self‐report (Scanlon et al. 2021). While a statistical interrogation of the prevalence of mental health disorders was not part of this study's design, fieldwork observations were not inconsistent with findings that cohousing communities have lower levels of depression, anxiety, compulsive and eating disorders than traditional neighbourhoods (Schetsche et al. 2020).

Taken together, the findings demonstrate the importance of locating mental health and wellbeing within a whole community rather than merely within individuals themselves. The largely positive impacts outlined by participants appear to be made possible due to the community's physical design, values‐based self‐governance, and culture of sharing and mutual support. Given concerns about rising rates of mental health disorders (Dykxhoorn et al. 2024), particularly among young people (McGorry et al. 2024), housing may offer a promising target for intervention (and prevention) in addition to existing service provision. A psychological framing of housing has implications for policies that seek to address loneliness and social isolation, at a time when many high‐income countries are looking for ways to boost homebuilding (Ministry of Housing Communities and Local Government 2024).

There are additional implications in the findings for psychological practitioners, who may wish to consider community connection and housing as part of a more holistic form of mental health assessment. The findings in this regard are consistent with the broadly positive preliminary analyses of the impacts of ‘social prescribing’ – a programme aimed at connecting people with chronic mental health difficulties with local community groups (Drinkwater et al. 2019; Morse et al. 2022).

### 4.2 Limitations of Cohousing for Mental Health and Well‐Being

While the present study provides broad support for the impact of cohousing on mental health and well‐being, it is important to note that there are also major challenges for residents. Extensive observations and discussions with interviewees were conducted regarding interpersonal conflict, workshare tensions, and the pressures of a dominant community culture. There is very little existing literature on conflict and disagreement in cohousing; most studies focus on the apparent benefits of community life (Carrere et al. 2020; Warner et al. 2024), and so this contribution is significant. Structured conflict‐resolution mechanisms appear to be particularly important for addressing disagreements occurring within cohousing, such as tensions around active contribution or disputes within consensus decision‐making. This is consistent with literature exploring the emergence of power imbalances within ‘structureless’ groups (Freeman 1972) and with research indicating that collective decision‐making is most successful in circumstances where structured communication takes place and when there are certain conditions, such as an expectation that group members will work within a consensus process (Renz 2006).

It was noted during fieldwork that the residents of Poplar Grove are similar in many demographic categories, despite efforts to recruit a more diverse membership, and that the community's functionality is guided by a set of visions and values that all members must subscribe to. This raises questions about whether some degree of cultural homogeneity is necessary in a cohousing context. Research from related fields indicates a positive association between neighbourhood social cohesion and homogeneity (Cheung and Leung 2011), but other studies of immigrant communities suggest that while demographic differences can be initially detrimental to trust and cooperation, societies tend to overcome fragmentation by developing more encompassing identities (Putnam 2007). More research is needed to explore this question.

### 4.3 Strengths and Limitations

Strengths of this study include its novel ethnographically informed research design, which enables a detailed understanding of mental health and well‐being outcomes in cohousing and represents an alternative epistemological approach to most mainstream research methods. The findings have implications for policy and practice that are clear and actionable. Additionally, this study breaks new ground in developing a better understanding not just of the apparent benefits of cohousing for mental health and wellbeing, but its negatives and risks, too.

There are limitations to this study due to its design. For instance, it was only possible to include a limited range of perspectives from one cohousing community, representing a self‐selected group of residents within Poplar Grove, but not including those who had left the community or chosen not to join. Further, necessitated by funding restrictions, this study employed a short duration of fieldwork and it is not possible to say how the findings would have been influenced by a different time‐period (such as during winter), or a longer duration. Future research may consider an alternative design to further explore the findings in this study, perhaps using experimental methods, a longitudinal approach, or a comparison study across multiple communities.

## 5 Conclusion

This ethnographically informed qualitative study explored how residents of a cohousing community in the United Kingdom understand and experience the impact of community living on their mental health and well‐being. It is the first known study to use ethnographic methods to study mental health and wellbeing in an intergenerational cohousing community and finds a broad range of positive impacts across the lifespan cited by interviewees and observed during fieldwork, and contributes towards a better understanding of the limitations of community membership and the mechanisms that help residents deal with conflict and tension. Findings highlight the importance of a psychological framing of housing, with implications for public policy and clinical practice.

## Funding

The authors have nothing to report.

## Ethics Statement

Ethical approval was granted by the University of Manchester Research Ethics Committee.

## Conflicts of Interest

The authors declare no conflicts of interest.

## Supporting information

Appendix A.

## Data Availability

The data that support the findings of this study are available from the corresponding author upon reasonable request.
