# Supplementary material for: Mental Health and Wellbeing in Cohousing Communities: An Ethnographically Informed Approach
Source: J Community Psychol. 2026 Apr 9;54(3):e70100. doi: 10.1002/jcop.70100 (PMC13077417; doi:10.1002/jcop.70100)
Supplement: Supplementary file 1 — Appendix A. [file JCOP-54-0-s001.docx]

Appendix A: Supporting Quotes

| **Theme** | **Additional Supporting Quotes** |
| --- | --- |
| **1) Social Pollination: How a Community Protects** | |
| 1.1 Social Support Across the Lifespan | One field notes extract provides an insight into how events promote social support: “This evening I helped cook a communal meal. About fifteen people attended (approximately half the adult community), and sat talking for nearly two hours. It was exceptionally intergenerational and inclusive, with the youngest 18 years of age and the oldest nearly 90. We sat around a long table in the common house. I noticed people talking about personal things, hobbies (cycling) and politics, including with the eldest member, who had memory problems. There was a moment of congratulations for the youngest adult member, who is sitting A-Levels and has just been given an unconditional offer to a university. The meal was paid for by members with tokens, representing £3 each, and accounting was made by the cook about what ingredients were used and who had contributed. Leftovers were split among those who needed/wanted. Someone volunteered to clean up.” (28/05)  One interviewee described the differences in social support in a close-knit community compared with a conventional neighbourhood:  “You wouldn't have a neighbour that knew that much about you probably in normal circumstances even if they did care about you, they wouldn't have that insight into your life. And I do think we've gone through a lot together like we've we've seen each other go through things, we've seen each other get older and have kids you know and lose babies and like have really sad things like lose parents and you know really big markers of your life.” (Interviewee 7) |
| 1.2 It Takes a Village | Field notes illustrate how the presence of multiple responsible adults, often also parents themselves, affected the daily experience of parenting:  “One of the two I was talking with is a mother of a four-month-old baby, who was asleep at the time. At one point she was called away by another child and left her sleeping baby with the resident I was speaking with, whose teenage children were also buzzing around in and out of conversation and activity. Three or four children varying by at least a decade in age, were cycling around on bikes, whizzing down the pathways between houses, until they eventually disappeared to play at one of their parents’ houses.” (27/05)  One interviewee described what it was like to get divorced while in the community setting and to seek support from neighbours: “We had immediate support from people from the community, that pick up the kids from school, take them to school, you know, without, and we know that they will be safe here, because they also have help from other members of community. And yeah, these in terms of hours, mental health, and all these kind of things, worries was really reduced, you know, completely. Yeah, because we, when we knew we knew that we could rely on our neighbourhood.” (Interviewee 4) |
| 1.3 When Things Get Hard | One interviewee disclosed struggles with Seasonal Affective Disorder and said that the community was very understanding: “I felt good about being able to make people aware of it. I think that was a beneficial thing. I didn't feel like that I had to hide and pretend that I wasn't getting depressed.” (Interviewee 3)  Others spoke of practical and emotional support from others: “It's just amazing how sort of the, you send a text you go: 'help,' and someone comes to your door and brings you food, and medicines, and will be like, 'I'm at the shops, what do you need?'” (Interviewee 1) |
| **2) Finding Your Tribe: Meaning and Purpose in Cohousing** | |
| 2.1 Building and Belonging | A review of community documentation illustrates the organised way that Poplar Grove sought to apply an agreed values system to decision-making. An extract: “We are people-led rather than institution-led, and focus on the needs of members rather than those of governments or businesses. We do this in a horizontal way, with task teams accountable to the whole membership, and important decisions made by consensus.” (Vision and Values Documentation) |
| 2.2 It Takes a Certain Kind of Person | One resident said that it can be helpful when residents are similar in outlook: “It makes it easier that we're aligned on lots of things. I have a dear friend whose critique of us is that he describes it is choosing to be living in a rarefied environment, where we choose our neighbours to be like us, rather than on a regular street where you're forced to live next to your neighbours and find meaning wherever they are…I do recognise that that we're quite a self-selecting group of largely middle-class people, I suppose.” (Interviewee 9)  Some residents said that the community recognised that one’s ability to contribute might fluctuate, even though it was an expectation that all residents would try: “It's recognising that everyone's circumstances, and it changes from week to week, year to year. But at least it's putting down what we ask of each other. But also, one of the keys to it is an opportunity to opt out. For whatever reason. We give some examples of the reasons why people may opt out. But if someone says, I'm going to opt out for the next three months of A B and C, then they can do that.” (Interviewee 3) |
| **3) Deconstructing Defensible Spaces** | |
| 3.1 The Sum of the Parts | Many residents said they learned from and were inspired by each other: “I've learned to appreciate more things and been surrounded by a lot of people with a lot of skills and knowledge and interests. It's just so, I find it so interesting. I may never want to do some of the things that some of my neighbours do, but I love hearing about how different people live.” (Interviewee 3)  Several people said that private spaces were also essential to wellbeing:  “I wouldn't want to live in a community if I didn't have my own kitchen and bathroom and my own front door. Because that I think that's really important. And I think I've I value I spend more time on my own now and value coming home and just being quiet and just doing my thing, and then engaging with things in the community when I want to.” (Interviewee 2) |
| 3.2 The Sharing Economy | For some residents, flexibility in affordability and the ability to move dwelling within the community allowed families to stay together even when parents split up: “We are looking really after those that need it. This is all about the community is looking for each other, you know, looking for each other's needs to facilitate.” (Interviewee 4)  However, some argued that there is still an obligation to pay a minimum amount to community upkeep and the mortgage, which could be seen as a financial burden: “If you're unable to pay, you're not able to claim benefit to pay towards your equity…ultimately, if you lost your job and was after a period of time was unable to get other employment, then you wouldn't be able to continue to live [here]. So in that sense, there isn't that economic security if you think about it.” (Interviewee 3) |
| **4) Conflict and Tension in a Social World** | |
| 4.1 The Burden of Social Pressure | Some residents described a difficult mental load from not being able to switch off from community obligation: “One of the ways I disconnect from my day job is that I'm not physically there…therefore don't think about it, or not very much. But when you've got [Poplar Grove] work that you haven't done, or you know, something that you're thinking, I'm gonna have to go work that one out, and you don't get that physical distance from it. And some people may just be able to switch that off. But other people may not. Other people can't. And then that can create quite a mental load really.” (Interviewee 6)  Others said they could self-censor when feeling under pressure or when decisions take too long through consensus processes: “So that is one of the weaknesses of consensus is it's I give up, self-censor, that is never going to happen, so I'm not really going to talk about it. And that suppression can be oppressive.” (Interviewee 9)s |
| 4.2 Tearing the Social Fabric… | One historic disagreement in the community had raised tensions between families to a degree that upset individuals and caused permanent ruptures: “That got as, yeah, spiky as more spiky than any anything else that has happened to me here and I was really upset about it. And we kind of did repair it, but I'd say that there's a certain amount of bad blood.” (Interviewee 7)  Others said that tension between other residents also affected them even when they weren’t involved: “Personally, I don't handle conflict very well. I tend to be someone who would avoid conflict in situations as opposed to face them. So when there's has been minor conflict. I have occasionally had a sleepless night over that. That's...they're quite rare, but I don't they do affect me when they happen.” (Interviewee 3) |
| 4.3 …And Learning How to Sew | The community conflict resolution agreement outlines “respect” and “a healthy, safe place” as core community values. It states: “we have all agreed to try to live together in a manner which fits with our values and vision. Our values, and the attitudes implicit in them, are in themselves conflict-reducing.” The agreement sets out stages to address conflict: 1) residents attempt to resolve the problem directly by themselves, 2) support is solicited from a mutually-accepted member to mediate, 3) to seek external facilitation or mediation if necessary, 4) residents can be expelled from the community, but only as a last resort under specific conditions. (Conflict Resolution Community Agreement)  Residents tried to think of disagreements as being about issues, rather than about people: “I think we are pretty good on the whole, collectively, in weathering disagreements over policy or practice or, you know, generally speaking, and others may tell you differently, but I think we're pretty good at saying: well, that was a disagreement over an idea or a policy or a way of doing things. It wasn't a disagreement with that person.” (Interviewee 3)  Fieldnotes illustrate an example of how disagreements may lead to inaction: “The conversation went on for about 15 minutes and there was an air of tension in the room – clearly some people felt this was a waste of time and not the point of the meeting, and that a disproportionate amount of time was being spent on this issue. Others felt it needed to be resolved today and should have been easier to do that than it seemed to be. I was also aware that possibly people were aware of my presence and what this example was saying about their process. Eventually it was proposed that the money be left in the budget and people would decide later how and whether it should be spent.” (05/06) |
